# PAN RNA: transcriptional exhaust from a viral engine

**DOI:** 10.1186/s12929-020-00637-y

**Published:** 2020-03-07

**Authors:** Mel Campbell, Yoshihiro Izumiya

**Affiliations:** Department of Dermatology and UC Davis Comprehensive Cancer Center, University of California Davis School of Medicine, 4645 2nd Avenue Research III Room 3100, Sacramento, CA 95817 USA

**Keywords:** Non-coding RNA, Herpesvirus, eRNAs, Chromatin looping, RNA pol II recruitment

## Abstract

Kaposi’s sarcoma-associated herpesvirus (KSHV), also designated human herpesvirus 8 (HHV-8), has been linked to Kaposi’s sarcoma, as well as to primary effusion lymphoma (PEL), and a subset of multicentric Castleman’s disease. KSHV genomes are maintained as episomes within infected cells and the virus exhibits a biphasic life cycle consisting of a life-long latent phase during which only a few viral genes are expressed and no viral progeny are produced and a transient lytic reactivation phase, in which a full repertoire of ~ 80 lytic genes are activated in a temporally regulated manner culminating in the release of new virions. Lytic replication is initiated by a single viral protein, K-Rta (ORF50), which activates more than 80 viral genes from multiple resident viral episomes (i.e.*,* viral chromosomes). One of the major targets of K-Rta is a long non-coding nuclear RNA, PAN RNA (polyadenylated nuclear RNA), a lncRNA that accumulates to exceedingly high levels in the nucleus during viral reactivation. K-Rta directly binds to the PAN RNA promoter and robustly activates PAN RNA expression. Although PAN RNA has been known for over 20 years, its role in viral replication is still incompletely understood. In this perspective, we will briefly review the current understanding of PAN RNA and then describe our current working model of this RNA. The model is based on our observations concerning events that occur during KSHV lytic reactivation including (i) a marked accumulation of RNA Pol II at the PAN promoter, (ii) genomic looping emanating from the PAN locus, (iii) interaction of a second viral lytic protein (ORF57) with K-Rta, PAN RNA and RNA Pol II, (iv) the essential requirement for PAN RNA expression *in cis* for optimal transcriptional execution needed for the entire lytic program, and (v) ORF57 recruitment of RNA Pol II to the PAN genomic locus. Together our results generate a model in which the PAN locus serves as a hub for sequestration/trapping of the cellular transcriptional machinery proximal to viral episomes. Sequestration at the PAN locus facilitates high levels of viral transcription throughout the viral genome during lytic replication. ORF57 acts as a transcription-dependent transactivator at the PAN locus by binding to both Rta and PAN to locally trap RNA Pol II. The resulting accumulation of high levels of nuclear PAN RNA created by this process is an inducible enhancer-derived (eRNA) by-product that litters the infected cell nucleus.

## Background

Herpesviruses are large, enveloped, double-stranded DNA viruses that infect a wide range of species. These viruses are classified into three subfamilies: *Alphaherpesvirinae*, *Betaherpesvirinae*, and *gammaherpesvirinae* [1]. The establishment of latency and life-long persistence within the host is a hallmark of herpesvirus infection. During infection, it has been found that herpesviruses generate a variety of non-coding RNAs, including lncRNAs, that have been ascribed a variety of roles in the viral life cycle. Table 1 summarizes some of the lncRNAs found among each family of herpesvirus. A detailed description of herpesvirus lncRNAs is beyond the scope of this review, but several recent reviews are available on this subject, both herpesvirus-specific or more broad overviews of viral lncRNAs [26–29].

**Table 1:** Herpesvirus lncRNAs

Virus and LncRNA Designation	Herpesvirus group	Acryonym	Transcript Size kb	Proposed functions	References
Herpes simplex type 1 (HSV-1) Latency Associated Transcripts	Alpha	LATs	8.3 6.3 2.0 1.5 + other species	Multiple controversial roles in virus reactivation efficiency, latency establishment, miRNA precursor	[2, 3]
Marek’s Disease Virus (MDV) Latency Associated Transcripts and MDV small RNAs	Alpha	LATs/MSRs	10 2.2 1.8 1.5 0.5 2.7	Antisense to ICP4	[4, 5]
Human Cytomegalovirus (hCMV) noncoding RNAs	Beta	RNA5.0 RNA4.9 RNA2.7 RNA1.2	5 4.9 2.7 1.2	Abundant, anti-apoptosis (2.7); interaction with cellular repressors (4.9)	[6–9]
Epstein-Barr Virus (EBV) BamHI-A rightward transcripts	Gamma	BARTs	Multiple species 4–8	Abundant, miRNA precursor	[10–12]
Kaposi’s Sarcoma-Associated Virus (KSHV) Polyadenylated Nuclear RNA	Gamma	PAN RNA	1.1	Facilitates viral late gene expression via nuclear RNA export; interaction with cellular epigenetic modifiers; interaction with viral latency protein	[13–18]
KSHV Antisense to latency transcript	Gamma	ALT	10	Unknown, Antisense to latency associated transcripts	[19, 20]
KSHV Transcript 3.0 KSHV Transcript 1.2	Gamma	T3.0 T1.2	3.0 1.2	Unknown, both transcripts antisense to ORF50 but not inhibitors of K-Rta expression, may encode small viral peptides	[21]
KSHV Transcript 1.4	Gamma	T1.4	1.4	Important for lytic DNA replication	[22, 23]
Rhesus Rhadinovirus (RRV) Polyadenylated Nuclear RNA	Gamma	RRV PAN RNA	1.3	Nuclear RNA export; can complement KSHV PAN RNA mutant	[18, 24]
Equine Herpesvirus 2 (EHV-2) Polyadenylated Nuclear RNA	Gamma	EHV-2 PAN RNA	1.9	Unknown; putative PAN RNA homolog with sequence homology to KSHV PAN expression and nuclear retention element (ENE)	[24]
Bovine herpesvirus 4 (BHV4) L1.7 RNA	Gamma	L1.7 RNA	1.5	Unknown, abundant, non-coding cytoplasmic RNA, some features similar to other PAN RNAs but has no ENE	[24, 25]

KSHV is a member of the gamma-herpesvirus family with a ~ 150 kb ds DNA genome. Recent comprehensive functional genomic approaches [30, 31] as well as earlier gene expression profiling of KSHV using real time PCR, oligonucleotide arrays, and northern blotting [19, 32] have revealed pervasive transcription of the KSHV viral genome, indicative of a complex viral transcriptome. This transcriptional complexity includes splicing events involving approximately one-third of the viral gene repertoire, sharing of single or alternative polyadenylation sites [33], and expression of non-coding RNAs including miRNAs [34, 35] and lncRNAs. To date, 16 potential lncRNAs have been reported by several groups using a variety of experimental approaches. Information regarding these lncRNAs have been compiled and superbly summarized by Schifano et al. [20]. KSHV lncRNAs have been characterized to varying levels of detail with the best studied species as the 1.1 kb polyadenylated nuclear RNA (PAN RNA), which is highly expressed during lytic replication. Although several early discoveries of regulatory RNAs such as H19 [36] and Xist [37, 38] existed at the time when PAN RNA was first described in 1996 [39, 40], its existence predates the widespread appreciation of lncRNAs that were subsequently uncovered later in the genomic era [41–44].

**Fig. 1:**
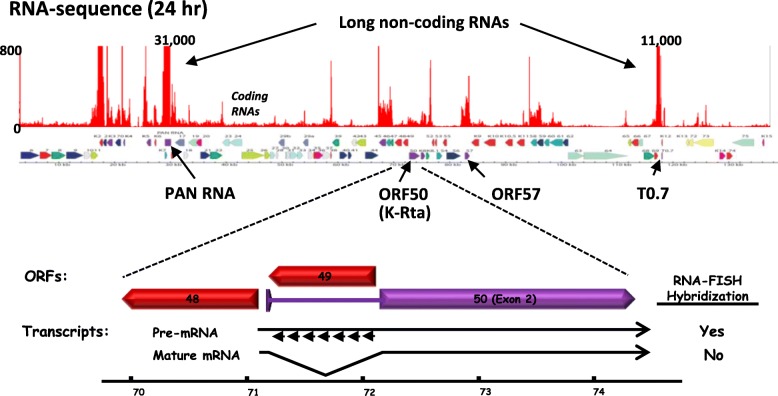
KSHV genome, early lytic gene expression and K-Rta RNA FISH. RNA-seq reads from KSHV-infected PEL cells at 24 h post-reactivation. Reads are positioned above a KSHV ORF map. Sequence reads for PAN RNA and another lncRNA (T0.7) predominate at this time point. The genomic position of the other viral components of the engine (ORF50 and ORF57) are indicated. The lower panel depicts the ORF50 locus in an expanded view showing the position of the RNA-FISH probes which detect unspliced K-Rta mRNAs and are used to image sites of viral transcription. Numbering beneath the transcripts (70-74) indicates viral map position in kb

PAN RNA is either expressed at low levels or non-expressed during latency but highly expressed during lytic reactivation with early kinetics [13, 30, 31] with PAN levels capable of reaching an estimated 1–5 × 10^5^ copies per cell in infected PEL cell lines [39, 45]. PAN RNA persists into the late lytic phase and is also packaged into virions [46]. The PAN RNA promoter is a direct target of K-Rta and PAN RNA expression is increased greater than 1000-fold during reactivation [45, 47, 48]. Consistent with the earlier abundance estimates, Arias et al. [30] RNA-seq results showed that during lytic replication, PAN RNA accounted for 65% of the KSHV reads at 8 h post-induction, and > 80% of the KSHV reads at 24-72 h post-induction. Please also see Fig. 1.

PAN RNA expression is an essential requirement for KSHV late gene expression and virus production [14, 15, 49]. Current research has proposed several functions for PAN RNA which can be broadly classified as chromatin associated or chromatin-independent. Chromatin-associated functions of PAN RNA link the lncRNA interaction with viral [15] or cellular [16, 17, 49] chromatin associated factors which facilitate late gene expression and virion production. These studies were grounded in the notion that PAN RNA, as a nuclear lncRNA, would exert its effects via interactions on chromatin, as had been reported for many cellular lncRNAs [50, 51]. However, when PAN RNA locales were examined in a PEL cell line using detailed fractionation protocols, Withers et al. [18] reported that the majority of PAN RNA was nuclear but did not associate with chromatin. This observation lends support to chromatin-independent functions of PAN coupling PAN RNA function to nuclear export of late viral mRNAs [14, 18]. Based on these reports, the exact role of PAN RNA in viral lytic replication is still unclear. Moreover, as mentioned above, the functional role for the presence of PAN RNA within virions is currently unknown.

## Model

Our model is based on studies combining molecular virology, capture Hi-C, RNA-seq, RNA-FISH and immunofluorescent imaging. We combine the following observations taken from the first ~ 24 h following reactivation to propose our working model as perspective, including (i) a marked accumulation of RNA Pol II at the PAN promoter (ii) genomic looping emanating from the PAN locus (iii) interaction of a second viral lytic protein (ORF57) with K-Rta, PAN RNA and RNA Pol II (iv) the requirement for PAN RNA expression *in cis* for optimal transcriptional execution needed for the entire lytic program and (v) ORF57 recruitment of RNA Pol II to the PAN genomic locus. We will detail each of these pieces of evidence in the sections that follow. The model is also inspired by the new convergence of cellular biophysics and transcription that has emerged from recent studies of subcellular compartments which suggest a role for liquid-liquid phase separation (LLPS) as a way to sequester specific proteins within both cytoplasmic and nuclear compartments that are not separated by membranes [52, 53]. These structures are proposed to be formed by multivalent protein–protein interactions mediated by intrinsically disordered regions (IDRs) within proteins that interact weakly and can be promoted through binding nucleic acids. Moreover, LLPS has also been associated with transcriptional condensates in vivo, consisting of large or clustered cellular regulatory sequences bound by protein complexes such as Mediator, interacting with promoters during transcriptional activation [54, 55]. IDR or low complexity (LC)-containing transcription factors bind regulatory sequences of DNA that can interact with the low complexity C-terminal domain (CTD) of RNA Pol II and stabilize its binding at transcriptional start sites to augment transcription initiation [54, 56] with regulation via CTD phosphorylation events [57, 58]. In many cases, enhancers are located at a distance from the promoters they regulate but these elements tend to abut in close proximity in 3D space during transcriptional activation [59, 60]. In contrast to typical enhancer (TEs), Young and colleagues coined the term ‘super-enhancer’ (SE) to characterize large genomic domains that differ from TEs [61]. SEs consist of large clusters of enhancer elements that are formed by the binding of key transcription factors and the Mediator coactivator complex [61]. Recent studies have linked SEs in the formation of LLPS condensates resulting in the convergence of SEs, LLPS, transcriptional regulation and genomic architecture. For example, transcriptional coactivators BRD4 and MED1 were found to be components of LLPS transcriptional condensates which co-localized with SEs [62] and LLPS-based transcriptional condensates have been proposed to explain the observation that a single enhancer can simultaneously co-activate multiple distinct promoters [60].

For KSHV, the expression of one viral protein (K-Rta; ORF50) is sufficient to trigger the entire viral lytic program. In addition to viral lytic transcriptional activity, additional changes are also observed on the KSHV genome during early reactivation including histone modifications [63] occupancy of chromatin modifiers [15, 64] and genomic contacts [65, 66]. Another interesting feature observed during the lytic switch is the formation of nuclear aggregates at sites of viral transcription. We termed these structures “transcription factories” based on the analogous structures previously observed in studies of eukaryotic gene expression (for review see [67]. Although cytoplasmic and nuclear viral transcription factories or replication compartments (RCs) have been known for many years (for DNA virus RC review see [68], the fundamental mechanisms behind their formation are still unclear. These KSHV structures, although incompletely defined in composition, are enriched for cellular RNA Pol II, viral transcripts, and replicating viral genomes [69]. Whether KSHV transcription factories are formed through LLPS is not known but some viral RCs possess several weak characteristics of liquid phase separation such as spherical shapes, the ability to fuse, and enrichment of viral proteins with IDRs. These RCs include those of herpes simplex virus type 1 (HSV-1 [70], rabies virus [71], and vesicular stomatitis virus (VSV [72]. However, a recent study also suggests that non-LLPS mechanisms facilitate in HSV-1 RC formation via preferential interaction of proteins with non-nucleosomal viral DNA available during viral replication reinforcing the notion that there are alternative pathways to compartmentalize proteins [70, 73]. Irrespective of the mechanism involved in factory formation, our central hypothesis is that protein/DNA/RNA aggregation induced by the recruitment of K-Rta triggers SE formation on viral chromatin, which includes both static and inducible genomic looping. These contacts facilitate the recruitment and entrapment of RNA Pol II and associated transcriptional machinery at specific viral loci such as the PAN RNA locus which then serves as a hub to support high levels of viral replication. Included in this network is the looping mechanism we described here to expedite high levels of PAN RNA transcription during the early stages of viral reactivation.

### Accumulation of RNA Pol II at the PAN promoter

**Fig. 2:**
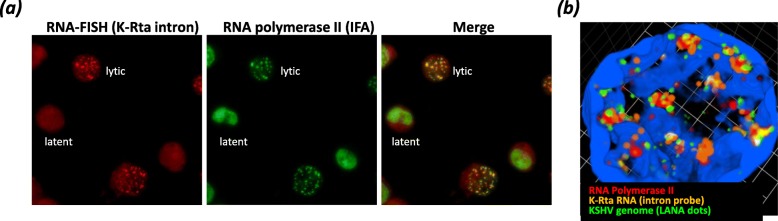
Visualization of KSHV transcription factories. **a** RNA-FISH (Red, K-Rta) and IFA (Green, RNA Pol II) colocalization (yellow) in a reactivated PEL cell population (24h) are shown. An example of cells scored as lytic or latent are indicated. **b** 3D view of KSHV transcription factory. Z-stack images were taken and 3D images with DAPI staining were constructed with Velocity imaging software (Quorum Technologies Inc., Canada). Green, LANA; Red, RNA Pol II; Orange, K-Rta RNA; Blue, DAPI

We recently adapted RNA-FISH approaches to study KSHV transcription in situ and to probe KSHV gene expression in the nuclei of infected host cells [69]. RNA-FISH probes were designed to hybridize to the K-Rta intronic region (Fig. 1) in order to specifically target the RNA transcripts immediately after being transcribed from viral episomes, but before spicing and nuclear export. Using RNA-FISH together with immunostaining of KSHV LANA protein, which constitutively binds to KSHV latent genomes, we observed significant heterogeneity in the response of individual KSHV episomes to stimuli within each reactivating cell. We found that not all episomes in a reactivating cell express viral RNA. Interestingly, those episomes responding to reactivation stimuli appeared to recruit molecules that formed large clusters at ~ 24 h post reactivation in PEL cells. We also observed that a significant fraction of cellular RNA Pol II was attracted towards KSHV genomes to form transcriptional factories which served to transcribe viral genomes and consequently led to an overall decrease in cellular gene expression activity in reactivating cells. The images also revealed that RNA Pol II aggregated in KSHV-reactivating PEL cells, but not in latent cells (Fig. 2a). Colocalization of LANA with RNA Pol II indicated clustering of RNA Pol II to the viral genome(s) during reactivation (Fig. 2b). The translocation of RNA Pol II was also confirmed by chromatin immunoprecipitation (ChIP), which demonstrated greater than 40-fold enrichment of RNA Pol II on the KSHV PAN RNA genomic region during reactivation (i.e., ChIP/Input ratios at the PAN locus relative to that of a cellular gene locus). We hypothesized that KSHV commandeers the cellular gene regulatory machinery to assemble viral genomic domains and recruit RNA Pol II, enabling highly efficient gene expression from appropriately structured viral episomes. This recruitment is especially robust at the PAN promoter during the initial stages of viral reactivation.

### Genomic looping from the PAN locus

Capture Hi-C analyses [74, 75] were used to examine KSHV genomic looping during latency and reactivation [66]. In these studies, we designed DNA oligonucleotide baits to comprehensively target the entire KSHV genome to allow for enrichment of KSHV-containing ligation products within each Hi-C library. Enrichment allowed us to document dynamic looping interactions between KSHV genomic fragments during the early stages of reactivation. We found that genomic loops were dynamically regulated during reactivation. K-Rta recruitment sites had a larger number of genomic interactions than non-K-Rta binding sites in a PEL cell line. The number of genomic loops increased especially among K-Rta recruitment sites during reactivation and were readily apparent near the PAN locus. These results suggest that spatial and temporal genomic interactions are important for tethering transactivator complexes at specific sites and are required for proper regulation of KSHV gene expression. The occurrence of inducible genomic loops at the K-Rta recruitment sites also suggests that the K-Rta protein complex may play a pivotal role in regulating inducible, higher-order genomic conformation.

### Cellular RNA polymerase II interacts with KSHV ORF57 protein on chromatin

While the mechanisms underlying genomic structural changes remain poorly understood, genomic 3D structure re-organization is known to be very important in many biological aspects such as tissue-specific gene expression [74, 76–79]. Taking advantage of the marked accumulation of RNA Pol II seen during KSHV reactivation (Fig. 2a, b), we isolated and identified proteins that may be responsible for such genomic assembly by utilizing **R**apid **I**mmunoprecipitation **M**ass spectrometry of **E**ndogenous protein (RIME) [80]. This method is well-suited to comprehensively identify interacting proteins within chromatin and transcription factor complexes on the genome. We examined protein partners of both K-Rta and RNA Pol II before and after reactivation. Non-induced samples served as a negative control for K-Rta, and control IgG was used to subtract non-specific interactions. Each RIME analysis was performed in duplicate, and more than 5 specific peptides identified in both of the duplicate samples were considered as specific interactions. The studies identified 9 viral and 87 cellular proteins as K-Rta-interacting proteins on the chromatin. The identified viral proteins are transcription regulators and/or DNA replication factors; these are ORF57, ORF59, ORF6, K-bZIP, vIRF1, ORF36, ORF17, ORF9, and ORF10. These cellular and viral proteins were also found in RIME studies with RNA Pol II. RIME positive hits are indicative that those molecules are localized in proximity to K-Rta and RNA Pol II on chromatin. Among them, ORF57 peptides were highly abundant (109 and 119 counts) in the duplicate samples. ORF57 proteins were also co-precipitated with K-Rta in RIME studies. ORF57 (also named mRNA transcript accumulation or Mta) is notable since it is known to bind PAN RNA and stabilizes the transcript [81, 82]. Accordingly, we focused on ORF57 in regulation of PAN RNA transcription with K-Rta. First, we examined the effects of ORF57-mediated increased PAN RNA stability on transactivation of distal promoters with reporter assays. We used reporter plasmids that contain various mutant PAN RNA expression cassettes and a luciferase coding sequence cloned downstream of an ORF16 promoter sequence. There is also an endogenous poly(A) signal inserted between the PAN RNA sequence and ORF16 promoter. We found that ORF57 enhanced K-Rta mediated ORF16 promoter activation, and that PAN RNA expression is necessary for downstream ORF16 promoter activation. Based on the reporter studies, we concluded that PAN RNA expression is required for the ORF57-mediated ORF16 promoter activation, because deletion of PAN RNA cassette diminished the synergistic effects with K-Rta. We further hypothesized that there should be commonly interacting cellular proteins that are brought in proximity to K-Rta by ORF57, and identification of those proteins may reveal molecular mechanisms of the synergistic activation of distal promoters. ORF57 protein interacts with a number of RNA binding proteins such as Poly(A) cleavage enzymes, proteins that function in DNA replication-dependent RNA regulation and splicing [81, 83]. Our RIME studies with ORF57 antibodies also indicated that ORF57 may preferentially recruit proteins that function at the 3′-UTR region of transcripts [82]. Noteworthy, the homologous immediate early protein of HSV-1 (ICP27) has also been reported to bind to RNA Pol II [84] and recruit RNA Pol II to viral replication sites [85].

### Essential requirement for PAN RNA expression *in cis* for optimal lytic transcription

We have previously developed a series of PAN mutants [66] using recombinant bacmids that contain the entire KSHV genome which can be engineered to contain a variety of desired mutations (designated as BAC16 [86];. Recombinant BAC16 vectors can then be transfected into cells and be stably maintained as latent viral episomes which can be reactivated to the lytic phase. Since the PAN promoter is a direct target of K-Rta, we mutated the K-Rta binding site in the PAN RNA promoter to study the effects of reduced PAN RNA expression within the context of the viral genome. The level of PAN RNA produced from PAN RNA promoter mutant (PAN Mu) was > 1000-fold less than cells containing wild-type BAC16. Using PAN Mu cells, we wished to determine if providing PAN *in trans* could rescue the defective lytic gene expression that exists in these cells. PAN Mu cells were transfected with a PAN expression vector 48 h prior to reactivation. This transfection raised the level of PAN RNA 3000-fold above the level present in reactivated PAN mutant cells transfected with an empty vector and was ~equivalent to the PAN RNA level obtained when wild-type BAC16 containing cells are reactivated. Despite this high level of ectopic PAN RNA complementation, lytic viral gene expression remained dramatically defective relative to reactivated cells containing wild-type BAC16. This lack of rescue suggests that activation of the PAN promoter and/or active transcription of PAN RNA is important for transactivation of many other viral lytic genes.

### ORF57 recruits RNA pol II to the PAN RNA genomic region

**Fig. 3:**
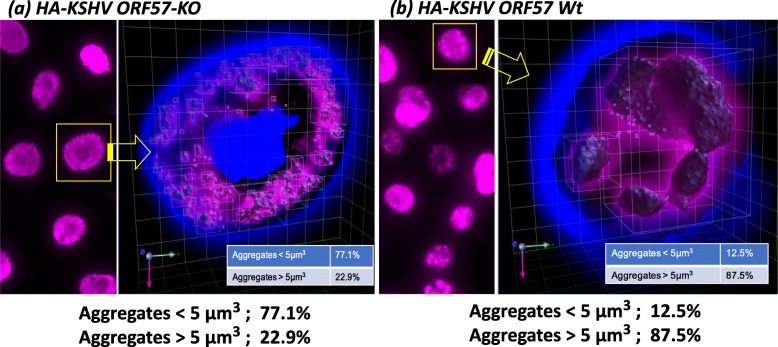
Cellular RNA polymerase II forms large peri-nucleolar aggregates after KHSV lytic reactivation. Fluorescence image stacks were imported, analyzed, and rendered in 3D using Volocity® Multi-Dimensional Imaging Platform (Quorum Technologies Inc., Canada). For each cell nucleus of interest, RNA Pol II aggregates were identified by fluorescence intensity over background and statistical information on aggregate volume, surface area, location, as well as minimum, maximum and average fluorescence signal were collected. **a** ORF57 knock-out (left) RNA Pol II is widely distributed throughout the nucleoplasm, with small aggregates (<5μm^3^) representing most of the total fluorescence. **b** With wild type KSHV reactivation (right) many cells exhibit prominent clustering of RNA pol II around a central void that likely represents the nucleolus. The distribution of RNA Pol II fluorescence measurably shifts towards large aggregates > 5μm^3^. (Images and analysis courtesy of Dr. Frank Chuang, UC Davis)

Enhancement of distal promoter activation strongly indicates that ORF57 not only stabilizes PAN RNA post-transcriptionally but may also contribute to increased recruitment of RNA Pol II either through interaction with K-Rta and/or nascent RNA molecules. To examine our model, we performed ChIP assays with recombinant BAC16 containing HA-tagged ORF57 wild-type or HA-tagged ORF-57Stop cells that produce a N-terminal truncated ORF57 protein. The results showed that in the absence of full-length ORF57 protein, recruitment of RNA Pol II to the PAN RNA coding region was significantly impaired. This is also in good agreement with RT-qPCR analyses, which showed decreased PAN RNA expression in the absence of ORF57. In addition, IFA visualization and quantification of RNA Pol II foci during reactivation demonstrated that ORF57 expression correlated with larger-sized aggregates (Fig. 3) Based on the facts that (i) ORF57 has global effects on KSHV gene expression, (ii) forms a complex with K-Rta and cellular poly(A) regulatory proteins, and (iii) increases occupancy of RNA Pol II at the PAN promoter, we hypothesize that ORF57 protein is a “ transcription-dependent transactivator”, whose function is to boost transcriptional activity by further recruiting RNA Pol II in the presence of active transcription, presumably through nascent RNAs. We also think that this function is not limited to PAN RNA and the PAN RNA promoter, since ORF57 is known to enhance several other viral transcripts, including coding RNAs. It is interesting to speculate that one function of ORF57 may be to contribute to the formation of LLPS by recruiting RNA binding proteins in a PAN RNA expression-dependent manner to facilitate the activation of other viral genes.

## Summary and concluding remarks

### A working model

**Fig. 4:**
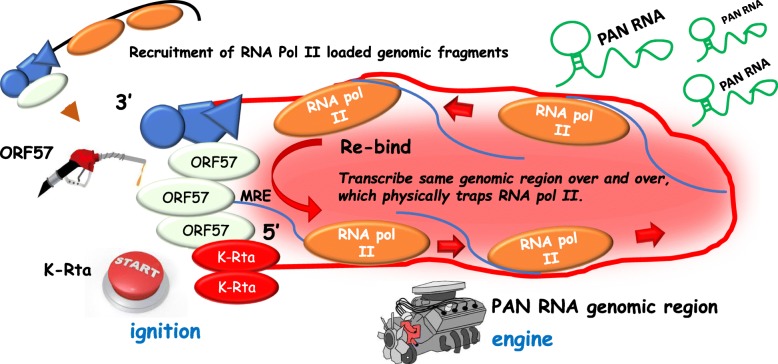
**Model**: Keeping resources for viral transcription in proximity by physically “trapping” RNA Polymerase II complex for effective viral gene expression. Nuclear PAN RNA as exhaust from the KSHV viral engine. Nascent PAN RNA accumulation is depicted as blue lines with RBPs (blue shapes) and ORF57 (light green ovals) bound. ORF57 binds to PAN RNA at multiple sites [106] including the Mta response element (MRE). The viral lytic engine starter motor is the viral transactivator K-Rta (red ovals), with the KSHV early gene product ORF57 serving as the fuel to drive the RNA Pol II rotor situated within the PAN RNA locus engine (red line). ORF57 is also a direct target of K-Rta and its lytic expression lags slightly behind that of K-Rta (not shown). Interactions between K-Rta, ORF57, RNA Pol II and nascent PAN RNA serve to keep RNA Pol II and associated transcriptional machinery trapped at the PAN promoter to facilitate high levels of PAN RNA. Excess PAN RNA production (green) accumulates as a by-product of running the high-powered viral engine. Running of the engine acts *in cis* drives expression of viral lytic genes along the entire KSHV episome. Hub formation via recruitment of additional RNA Pol II loaded genomic fragments (black line, upper left) is shown

Our working model is depicted in Fig. 4. ORF57 binds and recruits 3′-UTR genomic regions to the PAN RNA 5′-region through recognition of the PAN RNA MRE [ORF57/Mta responsive element (hairpin)] and/or physical interaction with K-Rta bound on a promoter. Increased numbers of genomic loops will then be formed at the PAN RNA genomic region through recruitment of 3′-UTR elements of distal genomic sites to the PAN RNA genomic region. This mechanism also facilitates recycling of assembled RNA Pol II complexes, accumulation of RNA Pol II at the genomic region, and increases accessibility to distal viral promoters in an active transcription-dependent manner. Closely clustered KSHV ORFs should also facilitate viral gene expression, because poly(A) sites and promoter regions are localized physically next to each other in the KSHV genome. Accordingly, K-Rta acts as the “starter”, ORF57 acts as fuel to feed K-Rta-mediated gene activation, and the non-coding RNA genomic locus functions as the engine to drive transcription of the entire KSHV genome with PAN RNA emitted as exhaust material.

Would the stabilized presence of the PAN locus 5′ and 3′ ends be sufficient to facilitate transcriptional re-initiation? There are several reports describing this type of interaction in yeast [87–89]. Indeed, the notion of contact between the rDNA promoter, enhancer and terminator units within the rDNA locus to ensure efficient RNA polymerase I recycling, a complex termed the “ribomotor”, was proposed more than 30 years ago [90] and experimental support for this model has been obtained for the murine rDNA system [91] and human mitochondrial rDNA system [92]. Yeast RNA polymerase III was reported to translocate from the terminator to the promoter of the same gene, and this event increased the rate of transcription re-initiation in vitro [93]. The role of the PAN RNA region can be envisioned as an active enhancer locus for the entire KSHV genome during lytic replication with transcription of this site generating PAN RNA as an enhancer RNA (eRNA). eRNAs are mainly derived from active enhancers and the transcription of enhancer regions into eRNAs has been correlated with increased transcription of adjacent genes [94–97]. Further evaluation of specific loci suggested that eRNAs themselves [98–100] or their transcription [101] are important for enhancer activity. As PAN RNA is extremely stable and polyadenylated [39] compared to eRNAs which are relatively unstable [102] and non-adenylated [96], PAN RNA does not exactly fit the definition of a canonical eRNA. However, the boundary between the definition of lncRNA and eRNA is not precise, as a lncRNA may function as an eRNA [101, 103]. Since previous reports [14, 15] have shown that RNase H-mediated knockdown of PAN RNA expression severely impairs late gene expression and virus production, PAN RNA itself and not PAN RNA transcription, per se, appears to be critical for enhancer activity. Although one group has reported that PAN RNA functions when supplied *in trans* [18], a requirement for the proper biogenesis of PAN RNA is consistent with our previous results [66] showing that ectopic expression of PAN RNA, via a plasmid vector which restored PAN RNA to levels observed in wild-type infected cells, did not rescue viral production from a PAN RNA mutant recombinant bacmid. Finally, although the title of our perspective relegates PAN RNA to waste material, a trans-acting function cannot be ruled out. How might appropriately generated PAN RNA function to support lytic gene expression in the context of our model? We have previous reported a large number of intragenic chromatin loops emanating from the PAN locus in both latent and lytic KSHV infected cells [66], thus PAN RNA may function via effects on KSHV genomic architecture. RNA-dependent functions for the boundary protein CTCF (CCCTC-binding factor) in chromatin looping have been reported recently [104, 105]. Further study is needed to understand the relationship between PAN RNA expression and KSHV genomic looping.

## Data Availability

The data reported in the current article have been published or are available from the corresponding authors on request.
